# Associations of Independent IL2RA Gene Variants with Intermediate Uveitis

**DOI:** 10.1371/journal.pone.0130737

**Published:** 2015-07-02

**Authors:** Ewald Lindner, Martin Weger, Navid Ardjomand, Wilfried Renner, Yosuf El-Shabrawi

**Affiliations:** 1 Department of Ophthalmology, Medical University Graz, Graz, Austria; 2 Clinical Institute of Medical and Chemical Laboratory Diagnostics, Medical University Graz, Graz, Austria; 3 Department of Ophthalmology, Klinikum Klagenfurt, Klagenfurt, Austria; Medical University Graz, AUSTRIA

## Abstract

**Purpose:**

The genetic background for the concomitance of uveitis and other autoimmune diseases remains elusive. Here the role of two IL2RA gene variants (rs11594656 and rs12722495) was investigated in intermediate uveitis and HLAB27 acute anterior uveitis.

**Materials and Methods:**

One hundred fifty-nine patients with HLAB27 acute anterior uveitis, 85 patients with intermediate uveitis, 138 HLAB27 negative controls and 100 HLAB27 positive controls were recruited for this case-control study. Main outcome measures were genotype distribution and allelic frequencies determined by polymerase chain reaction.

**Results:**

The frequencies of carriers of the minor allele at rs11594656 and rs12722495 were significantly different in patients with intermediate uveitis compared to HLAB27 positive and negative controls combined (p<0.05). For rs12722495 the minor G allele was protective (genotypic OR: 0.29 [0.12-0.69]), and for rs11594656 the minor A allele conferred risk (genotypic OR: 1.59 [1.09-2.32]). No significant differences in genotype distribution were found between patients with HLAB27 acute anterior uveitis and HLAB27 positive or negative control subjects.

**Conclusions:**

We found rs11594656 and rs12722495 to be associated with intermediate uveitis but not with HLAB27 acute anterior uveitis. The genetic heterogeneity found at the IL2RA locus could help explain patterns of concomitance with other autoimmune diseases.

## Introduction

The interleukin 2 receptor alpha (IL2RA) gene is a risk locus shared between various autoimmune diseases[1–3]. We have previously demonstrated an association of intermediate uveitis (IU) with rs2104286, a gene variant of IL2RA[4]. Since rs2104286 is strongly associated with multiple sclerosis (MS)[1], we suggested parallel pathogenic pathways between MS and IU. The rs2104286 gene variant is as well associated with other autoimmune diseases such as type 1 diabetes (T1D), but interestingly other single nucleotide polymorphisms (SNPs) associated with T1D show no association with MS suggesting allelic heterogeneity[5].

Intermediate Uveitis (IU) and Multiple Sclerosis (MS) are apparently related diseases. The majority of MS associated uveitis is IU[6–7]. Daclizumab, an antibody against IL2RA, which is used to reduce MS disease activity[8], has been shown to treat IU[9]. Despite that, most patients with MS do not develop IU and vice versa. Thus, IU might have pathogenic pathways that are either distinct or shared with other autoimmune diseases. Daclizumab has been discontinued by the manufacturer in 2009, which was due to diminishing market demands and not because of safety issues. Genetic findings, which highlight the role of IL2RA in IU(4), suggest to re-evaluate the role of daclizumab in terms of personalized therapy.

Interestingly, some alleles at IL2RA are disease-specific and some confer risk for one disease but protection from another. For T1D and MS several of these so called toggle SNPs have been found[10]. Rs11594656, a SNP in the IL2RA gene, has recently been found to be associated with MS and T1D. Importantly, this association at rs11594656 has been found to be independent from rs2104286[5, 11]. The A allele at rs11594656 confers risk to MS but protection from T1D suggesting that this allele acts as an “on switch” for MS or alternatively as an “off switch” for T1D. Given the concomitance of MS and IU we hypothesized that the A allele would confer risk to IU. As HLAB27 acute anterior uveitis (AAU) might be in a different class of autoimmune diseases, we did not expect an association with HLAB27 AAU.

Rs12722495 has been found to be associated with T1D.[11] In healthy donors a gene dosage effect on the IL2RA expression on CD4+ memory T cells has been demonstrated for rs12722495, but not for rs2104286[11]. Therefore it has been proposed that CD4+ memory T-cells are critical in the protection from T1D but not from MS, in which rs2104286 is the most strongly associated SNP. The found allelic heterogeneity as well as the corresponding phenotypical differences provide insight into the differing pathogenic pathways of autoimmune diseases.

Recently an autoinflammatory-autoimmune continuum has been proposed, which categorizes immune diseases according to their relation to the innate immune system on the one and the adaptive immune system on the other side[12]. There is some evidence suggesting that HLAB27 AAU and IU might be on different sides of the spectrum. Rosenbaum et al.[13] highlighted the role of the innate immune system showing that injection of lipopolysaccharide (LPS), which is the major component of the outer cell wall of Gram-negative bacteria, lead to HLAB27 AAU. Preceding infections play a role in disease development in conditions associated with HLAB27 AAU[14] and HLAB27 positive individuals show an impaired immune response upon microbial infection[15]. This suggests that autoinflammatory mechanisms are more important in HLAB27 AAU than in IU, which may be more influenced by autoimmune pathways. Variants in the IL2RA gene have been associated with impaired T regulatory cell function and thus may affect autoimmunity more than autoinflammation[16].

The purpose of our study was to investigate an association of rs11594656 and rs12722495 with IU and HLAB27 AAU. Furthermore, we wanted to highlight allelic heterogeneity at the IL2RA locus between various autoimmune diseases.

## Materials and Methods

The participants of this study were of European descent, living in the same geographical area and were seen at the local Department of Ophthalmology, Medical University of Graz, Austria. Written informed consent was obtained from the subjects after explanation of the nature and possible consequences of the study. The study was conducted according to the tenets of the Declaration of Helsinki (seventh revision, Fortaleza, 2013) und was approved by the ethics committee of the Medical University of Graz (IRB00002556).

The following data were obtained from all patients: gender, age at presentation, age at onset of uveitis, systemic disease association, number of flares, duration of flares, duration between flares, and prevalence of severe ocular complications. Ocular complications were defined as significant cataract (greater than or equal to 2+ opacity) or secondary glaucoma. The diagnosis of HLAB27 AAU or IU was based on the SUN criteria[17]. All patients suffering from HLAB27 AAU were examined for clinical and radiographic signs and symptoms of spondylarthropathy by a rheumatologist. Radiographs of the sacroiliac joints and the spine were made when patients had inflammatory back pain or at least other symptoms compatible with the presence of spondylarthropathy. MRI scan of the brain was obtained in all patients suffering from IU and examined for the presence of radiologic signs in accordance with a possible diagnosis of MS such as presence and distribution of white matter lesions. In case of neurological symptoms patients were examined by a neurologist. In these patients also lumbar puncture with testing for oligoclonal bands was performed.

As controls 149 random, unrelated, healthy individuals attending our department for reasons other than ocular inflammation were included. Exclusion criteria were any history of intraocular inflammation, arthritis, lower back pain, autoimmune diseases or malignancy. None of the controls showed any signs of past uveitis episodes (e.g. residual pigment on lens) in slit-lamp examination. The past medical history was collected following a routine questionnaire. Of course it cannot be ruled out that the controls will eventually develop autoimmune diseases or malignancies in the future. All control subjects were genotyped for HLAB27. Eleven HLAB27 positive controls, together with 89 HLAB27 positive healthy unrelated blood donors, whose DNA was provided by the Department of Blood Serology and Transfusion Medicine, served as the HLAB27 positive control group.

### Genetics

DNA was extracted from peripheral lymphocytes using the nucleic isolation kit: QIAamp DNA Mini and Blood Kit (QIAGEN; Netherlands) following the manufacturers protocol and stored at -20°C.

Genotype determination was performed using high-resolution melting curve analysis on the LightCycler 480 PCR system. The samples were amplified in duplicate 20μl reactions using the Light Cycler 480 High Resolution Melting Master kit (Roche Diagnostics, Vienna, Austria) and analyzed on a LC480 instrument I (Roche Diagnostics GmbH, Mannheim, Germany). The final reaction mixture contained 1x Master Mix, 3mM MgCl2, 4μM forward and reverse primer and 50ng of genomic DNA. For PCR the following cycling conditions were chosen: one cycle of 95°C for 10 minutes followed by 45 cycles of 95°C for 10 seconds, 60°C for 15 seconds and 72°C for 20 seconds. The amplicons were then denaturated at 95°C for 1 minute, cooled down to 40°C for 1 minute and then melted from 65°C to 95°C with 25 signal acquisitions per degree. To detect sequence variations the Gene Scanning Software version 1.5 (Roche Diagnostics GmbH, Mannheim, Germany) was used. Samples were automatically grouped because of their melting curves using the Auto Group mode.

### Statistics

Statistical analysis was performed using SPSS for Windows (release 15.0, SPSS Inc., Chicago, IL). Means were compared using Mann-Whitney test. Proportions of groups were compared by the χ2 test. Odds ratio (OR) and 95% confidence interval (95% CI) were calculated by logistic regression. The criterion for statistical significance was p≤0.05. P-values were adjusted using Bonferroni correction. Hardy-Weinberg equilibrium has been calculated using HW Diagnostics-Version 1.beta (Fox Chase Cancer Center, Philadelphia, PA). Linkage disequilibrium was calculated with Haploview version 4.2 (Broad Institute, Cambridge, MA). Statistical power was calculated using PS Power and Sample Size Calculation software Version 2.1.30[18].

## Results

Our study comprised 159 patients with HLAB27 AAU (71 female [44.7%]), 85 patients with IU (50 female [58.8%]), 138 HLAB27 negative controls (41 female [29.7%]) and 100 HLAB27 positive controls (51 female [51.0%]). The mean age was 44.8 ± 14.3 for patients with HLAB27 AAU, 31.2 ± 15.9 for patients with IU, 35.3 ± 12.5 for HLAB27 negative controls, and 38.2 ± 4.2 for HLAB27 positive controls. Since polymorphisms do not change during lifetime differences in age between the groups were tolerated.

Clinical characteristics of patients are shown in Table 1. Mean age of onset was 36.36 ± 14.17 years for HLAB27 AAU and 27.91 ± 14.59 years for IU patients. Two patients with IU had MS (0.8%). No association was found between the investigated gene variants and any of the listed ocular or systemic parameters.

**Table 1:** Baseline ocular and systemic parameters.

Patient characteristics	HLAB27 AAU	IU
	(n = 159)	(n = 85)
Mean age of onset ± SD (years)	36.36 ± 14.17	27.91 ± 14.59
Mean number of flares ± SD	7.26 ± 9.24	4.24 ± 7.17
Mean duration of flares ± SD (weeks)	4.63 ± 2.74	5.25 ± 6.39
Mean duration between flares ± SD (months)	22.69 ± 18.31	13.29 ± 12.10
One eye affected	90 (56.6)	31 (36.5)
Both eyes alternating	56 (35.2)	11 (12.9)
Both eyes concomitant	13 (8.2)	43 (50.6)
Secondary cataract	21 (13.2)	13 (15.3)
Secondary glaucoma	5 (3.1)	3 (3.5)
Ankylosing spondylitis	62 (39.0)	0 (0.0)
Juvenile idiopathic arthritis	1 (0.6)	0 (0.0)
Undifferentiated spondylarthritis	21 (13.2)	0 (0.0)
Reactive arthritis	5 (3.1)	0 (0.0)
Crohn´s disease	1 (0.6)	1 (1.18)
Psoriatic arthritis	13 (8.2)	0 (0.0)
Multiple sclerosis	0 (0.0)	2 (2.4)

HLAB27 AAU = HLAB27 associated acute anterior uveitis

IU = intermediate uveitis

SD = standard deviation

Values are n (%) unless otherwise indicate.

To rule out any possibility that a putative association between the investigated variants and HLAB27 AAU was solely due to linkage with HLAB27, the control group was separated into HLAB27 positives and HLAB27 negatives. No significant differences in genotype distribution were found between patients with HLAB27 AAU and HLAB27 positive or negative control subjects (p≥0.05; Table 2).

**Table 2:** Distribution of the investigated gene polymorphisms in patients and controls.

	HLAB27 AAU (n = 159)	IU (n = 85)	HLAB27 negative controls (n = 138)	HLAB27 positive controls (n = 100)	HLAB27 negative and positive controls combined (n = 238)
rs 11594656					
T/T	93 (58.5%)	31 (36.5%)	77 (55.8%)	56 (56.0%)	133 (55.9%)
T/A	57 (35.8%)	45 (52.9%)	46 (33.3%)	39 (39.0%)	85 (35.7%)
A/A	9 (5.7%)	9 (10.6%)	15 (10.9%)	5 (5.0%)	20 (8.4%)
T	243 (76.4%)	107 (62.9%)	200 (72.5%)	151(75.5%)	351 (73.7%)
A	75 (23.6%)	63 (37.1%)	76 (27.5%)	49 (24.5%)	125 (26.3%)
Allelic P	0.27*	0.01‡			
	0.81†				
Allelic OR (95% CI)	1.23 (0.85–1.78)*	1.65 (1.14–2.40)‡			
	1.05 (0.70–1.59)†				
Genotypic P	0.31*	0.02‡			
	0.82†				
Genotypic OR (95% CI)	0.83 (0.58–1.19)*	1.57 (1.08–2.30)‡			
	0.95 (0.63–1.45)†				
rs 12722495					
A/A	120 (75.5)	79 (92.9)	110 (79.7)	75 (75.0)	185 (77.7)
A/G	36 (22.6)	6 (7.1)	26 (18.8)	24 (24.0)	50 (21.0)
G/G	3 (1.9)	0 (0.0)	2 (1.4)	1 (1.0)	3 (1.3)
A	276 (86.8%)	164 (96.5%)	246 (89.1%)	174(87.0%)	420 (88.2%)
G	42 (13.2%)	6 (3.5%)	30 (10.9%)	26 (13.0%)	56 (11.8%)
Allelic P	0.38*	0.01‡			
	0.95†				
Allelic OR (95% CI)	0.80 (0.49–1.32)*	0.27 (0.12–0.65) ‡			
	0.98 (0.58–1.66)†				
Genotypic P	0.93*	0.01‡			
	0.81†				
Genotypic OR (95% CI)	0.98 (0.57–1.68)*	0.29 (0.12–0.70)‡			
	0.94 (0.53–1.63)†				

HLAB27 AAU = HLAB27 associated acute anterior uveitis

IU = intermediate uveitis

* compared with HLAB27 negative controls

^†^ compared with HLAB27 positive controls

^‡^ compared with HLAB27 negative controls and HLAB27 positive controls combined.

In IU a significant association was found for rs11594656 (p = 0.01, OR: 1.65 [1.14–2.40]) and rs12722495 (p = 0.01, OR: 0.27 [0.12–0.65]). The minor A allele at rs11594656 conferred risk to IU and the minor G allele at rs12722495 was protective. Observed genotype frequencies of both polymorphic markers were in accordance with the Hardy-Weinberg equilibrium (data not shown). Genotype distribution of control subjects were similar to those reported for other populations of European descent[5, 19]. The investigated polymorphisms were in low linkage disequilibrium (D´ = 0.45).

## Discussion

This is the first study to demonstrate an association of rs11594656 and rs12722495 with IU.

Functional studies aiming to connect risk alleles with immunophenotypes are critical to understand how genetic variants affect mechanisms of autoimmunity. A recent study demonstrated the influence of three IL2RA gene variants (rs12722495, rs2104286 and rs11594656) on surface expression in healthy individuals[11]. Individuals with protective alleles at rs12722494 showed higher mean IL2RA levels on CD4+ memory T cells compared to fully susceptible individuals. Furthermore, a gene dosage effect at rs12722495 was observed. Interestingly rs12722495 was associated with phenotypic changes in all three cell types (CD4+ memory T cells, naïve T cells and stimulated CD14+ CD16+ monocytes), while rs2104286 was correlated with only two phenotypic changes (in naïve T cells and stimulated CD14+ CD16+ monocytes). Since rs2104286 is the most strongly associated SNP in MS it has been proposed that CD4+ memory T cells are more critical in the protection from T1D than from MS. For rs12722495 we found an OR of 0.29 in IU patients. Therefore, we suggest that CD4+ memory T cells play an important role in IU as well.

We have recently demonstrated a protective effect of the minor G allele at rs2104286 in IU[4]. Rs2104286 lowers IL2RA on naïve T cells, thereby reducing the likelihood of activation. In contrast rs12722495 correlates with higher IL2RA levels on CD4+ memory cells, which are in vivo the primary source of IL-2 required for FOXP3+ Treg cells[20] thereby reinforcing self-tolerance.

Rs11594656 alone accounts for 6.6% of the total variance in soluble IL2RA concentrations and carriage of the minor A allele correlates with higher serum levels of sIL2RA[2].

The precise pathophysiological role of sIL2RA in the etiology of autoimmune diseases remains elusive. Elevated concentrations of sIL2RA were detected in several autoimmune conditions, including MS[21], SLE[22], Crohn’s disease[23], celiac disease[23], and RA[24]. Soluble IL2RA is able to bind and neutralize IL-2, which is necessary for the activation of CD4+ CD25+ Tregs. Although no significant difference in numbers of CD4+CD25^*high*^Tregs between controls and patients has been found in MS [25] or Grave´s Disease, the function of these cells was reduced and impaired[26]. Increased levels of sIL2RA could contribute to autoimmunity by compromising the functionality of Tregs.

In a Spanish population the two polymorphisms investigated herein as well as rs2104286 were not associated with endogenous non-anterior uveitis[27]. As the authors stated the power to detect an association with IU was limited in the stratified analysis. Different ethnicities as well could contribute to the conflicting results.

The allelic heterogeneity observed in this study between IU and HLAB27 AAU highlights the necessity of investigating each disease individually. We observed that the A allele at rs11594656 confers susceptibility to IU. The same allele confers susceptibility to MS but protection from T1D[5]. This finding presents add to our understanding of the commonality between MS and IU. Still not all patients with IU develop MS, so we presume distinct disease specific alleles and alleles shared with other autoimmune diseases. Here we demonstrated an association of IU with rs12722495, which is as well associated with T1D.

The following potential limitations should be kept in mind, when interpreting our results. First, only a small number of SNPs were investigated in the present study. So we cannot rule out, that a true causative variant is located somewhere else within the LD block, therefore sequencing of the IL2RA gene may reveal further associations of other IL2RA gene variants and IU risk. Second, as genetic polymorphisms have been shown to vary between populations, our findings do not necessarily apply to populations other than of European descent.

As rs2104286, rs11594656 and rs12722495 belong to 3 independent SNP groups(5, 11) our findings present novel and independent assocations of IL2 RA variants with IU in an Austrian population.
